# Pathological Mycology

**Published:** 1885-09

**Authors:** 


*Pathological Mycology*. By G. SIMS WOODHEAD and A. W. HARE. Section I.—Methods. Pp. 174. Edinburgh: Y. J. PENTLAND. 1885.

This work is intended as a practical guide to the examination and cultivation of micro-organisms, especially of those that are supposed to be connected with disease. So far, the authors have fully and satisfactorily succeeded in their purpose. There is no work in the English language which, in so small a space, gives such accurate and trustworthy accounts of the best methods of working at this difficult and delicate subject. There is little attempt at originality, either in method or in pursuit; the work simply represents the most approved practical plans of working at the subject in pathology and experiment. The selections have been made with the judgment and sincerity of full knowledge and honest discrimination; we are not worried either with the hobbies of laboratories, or with the abuse of investigators, and the whole subject is presented in a simple and workmanlike spirit, which is both soothing and satisfactory.

We must once again bestow a word of praise on the manner in which the publisher has done his work. The printing and binding are as usual excellent, but the illustrations are simply beyond all praise. We confess to an ignorance of the process whereby poly-chromic representations of great beauty and finish are introduced in the midst of the letterpress without apparently affecting the cost, while we heartily recognise its value for purposes such as are aimed at in this work.

